# CircRNA LOC729852 promotes bladder cancer progression by regulating macrophage polarization and recruitment via the miR‐769‐5p/IL‐10 axis

**DOI:** 10.1111/jcmm.18225

**Published:** 2024-03-20

**Authors:** Changming Dong, Pengyu Hui, Zhengqi Wu, Jianfeng Li, Xiaojun Man

**Affiliations:** ^1^ Department of Urology, China Medical University The First Hospital of China Medical University Shenyang Liaoning China; ^2^ Department of Urology The First Hospital of China Medical University Shenyang Liaoning PR China; ^3^ Department of Urology The Second Affiliated Hospital of Xi'an Medical University Xi'an Shaanxi China

**Keywords:** autophagy, bladder cancer, CircRNA, IL‐10, M2 polarization, macrophage

## Abstract

Circular RNAs (circRNAs) function as tumour promoters or suppressors in bladder cancer (BLCA) by regulating genes involved in macrophage recruitment and polarization. However, the underlying mechanisms are largely unknown. The aim of this study was to determine the biological role of circLOC729852 in BLCA. CircLOC729852 was upregulated in BLCA tissues and correlated with increased proliferation, migration and epithelial mesenchymal transition (EMT) of BCLA cells. MiR‐769‐5p was identified as a target for circLOC729852, which can upregulate IL‐10 expression by directly binding to and suppressing miR‐769‐5p. Furthermore, our results indicated that the circLOC729852/miR‐769‐5p/IL‐10 axis modulates autophagy signalling in BLCA cells and promotes the recruitment and M2 polarization of TAMs by activating the JAK2/STAT3 signalling pathway. In addition, circLOC729852 also promoted the growth of BLCA xenografts and M2 macrophage infiltration in vivo. Thus, circLOC729852 functions as an oncogene in BLCA by inducing secretion of IL‐10 by the M2 TAMs, which then facilitates tumour cell growth and migration. Taken together, circLOC729852 is a potential diagnostic biomarker and therapeutic target for BLCA.

## 1 INTRODUCTION

Bladder cancer (BLCA) is the ninth most commonly diagnosed malignancy and, respectively, ranks seventh and ninth among males and females in terms of incidence.^1^ Despite recent advances in treatment strategies, the prognosis of BLCA patients is still not optimistic, with a recurrence rate of up to 70% in 5 years.^2^ Furthermore, the molecular mechanisms underlying the development of BLCA remain uncertain. Therefore, there is an urgent need to identify novel biomarkers of BLCA in order to formulate therapeutic strategies.

Circular RNAs (CircRNAs) are endogenous RNAs with closed conserved loop structures lacking the 3′ tail and 5′ cap and are formed by splicing of a precursor RNA.^3^ Due to their conserved loop structure, circRNAs are more stable than linear RNAs. In addition, circRNAs have been detected in the plasma, exosomes and urine. Given their stability and tissue specificity, circRNAs are increasingly being studied as diagnostic biomarkers and therapeutic targets.^4^ Furthermore, multiple circRNAs have been identified that regulate genes involved in tumour proliferation, development, angiogenesis, metastasis and invasion^5^ by directly binding to micro RNAs (miRNAs) through miRNA response components (MRES).^6^ For instance, the circVDAC3 is overexpressed in colorectal cancer (CRC) tissues and inhibits tumour progression via the miR‐942‐5p/BATF2 axis.^7^ Furthermore, circVDAC3 is also overexpressed in the plasma of CRC patients, and high levels have been detected in the tumour tissues of lung cancer patients in Xuanwei.^8^, ^9^

According to the human reference genome (GRCh37/hg19)，circLOC729852 (hsa_circ_0001676) was located at chr7:7826418–7841374+, originated from its host gene LOC729852(UMAD1) and transcribed from its exon 4. LOC729852 has been widely discussed in interstitial lung diseases complicated with rheumatoid arthritis, especially Alzheimer's disease. However, the role of circLOC729852 in bladder cancer and its molecular mechanism are still unclear.^10^, ^11^, ^12^

Autophagy is a catabolic process that provides energy to cells during stress conditions such as starvation or hypoxia by degrading misfolded proteins and damaged organelles in the lysosomes.^13^ Aberrant autophagy has been linked to cancer development and chemotherapy resistance.^14^ circRNAs importantly regulate the cellular processes such as cell proliferation, differentiation, apoptosis and autophagy.^15^, ^16^ Both circRNAs and autophagy are involved in the pathogenesis of metabolic diseases.^17^ Recent studies have shown that circRNAs regulate autophagy through different molecular pathways, which in turn affect the progression of diseases.^18^ Previous studies have shown that circRNAs mediate autophagy in cancer cells.^19^, ^20^ For instance, circ0007813 promotes BLCA by regulating autophagy in the tumour cells through the miR‐361‐3p/IGF2R pathway.^21^ However, the autophagy‐related circRNAs have not been fully explored in BLCA.

Dysregulated circRNAs play pivotal roles in diseases, particularly in tumour development, influencing cell proliferation, apoptosis and metastasis.^22^, ^23^ Most importantly, circRNAs have emerged as potent modulators of the tumour microenvironment (TME) and have a prospective role in tuning immunotherapeutic regimens' efficiency and outcomes.^24^, ^25^, ^26^ TME is a key factor regulating tumour growth, immunosuppression, drug resistance and metastasis.^27^, ^28^, ^29^ Apart from the tumour cells, the TME typically includes fibroblasts, stromal cells, endothelial cells, immune cells, chemokines and cytokines.^30^ The tumour‐associated macrophages (TAMs) are the dominant infiltrating immune cells in most tumours^31^, ^32^ and can be polarized into the M1 and M2 phenotypes by the cytokines in the TME.^33^ The unpolarized M0 TAMs can be induced to the M1 phenotype by interferon‐γ (IFN‐γ), while IL‐13 and IL‐4 induce M2 polarization.^34^, ^35^ The M1 TAMs are potent inflammatory cells, and the M2 cells promote tissue remodelling and repair, as well as tumour progression.^33^ Studies show that increased tumour infiltration of M1 macrophages is associated with better prognosis, while greater abundance of M2 TAMs has the opposite effects.^36^, ^37^, ^38^ In most tumours, TAMs tend to be of M2 polarity, suggesting that the infiltrating M2 TAMs are a promising therapeutic target.^39^ The infiltration of M2 TAMs can be controlled by inhibiting recruitment or activation, direct elimination of the cells or reprogramming to the M1 phenotype.^40^, ^41^

In this study, we screened for aberrantly expressed circRNAs in BLCA using transcriptomics data and found that circLOC729852 (circBase number: hsa_circ_0001676) is highly expressed in BLCA tissues and correlates with the clinicopathological stage. Mechanistically, circLOC729852 facilitates the recruitment and M2 polarization of TAMs by upregulating IL‐10 via activation of the JAK2/STAT3 pathway, and the M2 TAMs promote BLCA progression by inducing IL‐10 expression in the tumour cells. Thus, circLOC729852 is a promising therapeutic target for BLCA.

## 2 MATERIALS AND METHODS

### 2.1 Screening of circRNAs

The differentially expressed circRNAs in bladder urothelial carcinoma were identified by screening the GSE92675 and GSE147984 dataset in the GEO database.^42^

### 2.2 Cell lines

T24, UM‐UC‐3 and SV‐HUC‐1 cells were purchased from Xinzhou Biotech Company in Zhongqiao, Shanghai. The cells were cultured in RPMI‐1640 medium or DMEM supplemented with 10% serum (Sangon Biotech) at 37°C under 5% CO_2_.

### 2.3 Cell transfection

BLCA cells were seeded in six‐well plates and transfected with circLOC729852 siRNAs or negative control (NC) (50 pmol/well) when 60% confluent using 6 μL Lipofectamine 3000 (Thermo Marshall Tech). The shRNA sequences are listed in Table 1.

**TABLE 1:** The sequence of siRNA and primers.

Sequence type	Sequence (5′‐3′)
NC	
Top strand	CACCAACCAACCTTTGGAGAGTGAACGAATTCACTCTCCAAAGGTTGG
Bottom strand	AAAACCAACCTTTGGAGAGTGAATTCGTTCACTCTCCAAAGGTTGGTT
sh‐circ‐1	
Top strand	CACCG*TGGAGAGTGAAGAGAGAACCTCGAAAGGTTCTCTCTTCACTCTCCA
Bottom strand	AAAATGGAGAGTGAAGAGAGAACCTTTCGAGGTTCTCTCTTCACTCTCCAC*
sh‐circ‐2	
Top strand	CACCG*CTTTGGAGAGTGAAGAGAGAACGAATTCTCTCTTCACTCTCCAAAG
Bottom strand	AAAACTTTGGAGAGTGAAGAGAGAATTCGTTCTCTCTTCACTCTCCAAAGC*
miR‐769‐5p	
Inhibitor	ACUCUGGAGACCCAAGACUCG
mimic	UGAGACCUCUGGGUUCUGAGCU‐
sh‐IL‐10‐1	
Top strand	CACCGCTGGACAACTTGTTGTTAAACGAATTTAACAACAAGTTGTCCAGC
Bottom strand	AAAAGCTGGACAACTTGTTGTTAAATTCGTTTAACAACAAGTTGTCCAGC
sh‐IL‐10‐2	
Top strand	CACCGCCTAACATGCTTCGAGATCTCGAAAGATCTCGAAGCATGTTAGGC
Bottom strand	AAAAGCCTAACATGCTTCGAGATCTTTCGAGATCTCGAAGCATGTTAGGC
hsa‐miR‐27a‐5p	
Forward	GCGAGGGCTTAGCTGCTTG
Reverse	AGTGCAGGGTCCGAGGTATT
hsa‐miR‐3688‐3p	
Forward	CGCGTATGGAAAGACTTTGC
Reverse	AGTGCAGGGTCCGAGGTATT
hsa‐miR‐4283	
Forward	GCGCGTGGGGCTCAGC
Reverse	AGTGCAGGGTCCGAGGTATT
hsa‐miR‐6790‐3p	
Forward	GCGACCTCGGCGACCC
Reverse	AGTGCAGGGTCCGAGGTATT
hsa‐miR‐6821‐3p	
Forward	GCGTGACCTCTCCGCTCC
Reverse	AGTGCAGGGTCCGAGGTATT
hsa‐miR‐769‐5p	
Forward	CGCGTGAGACCTCTGGGTTC
Reverse	AGTGCAGGGTCCGAGGTATT
hsa‐miR‐6789‐5p	
Forward	AGGGGCGTCCCGGGCG
Reverse	AGTGCAGGGTCCGAGGTATT
U6	
Forward	CTCGCTTCGGCAGCACA
Reverse	AACGCTTCACGAATTTGCGT
circMCTP2	
Forward	GGTCTCTCATTCCCCACCAT
Reverse	CCCCTTTCTACCTCCTCTTCC
circSLAMF6	
Forward	GTCCTGAGCAAATTTGGAGCA
Reverse	CCTAAGCTAGTCCCTCAGGT
circLOC729852	
Forward	AGCAAAGAATGACAGCAAGAGG
Reverse	CCTTCTCTTCTCCCACTCCC
IL‐10	
Forward	GACTTTAAGGGTTACCTGGGTTG
Reverse	TCACATGCGCCTTGATGTCTG

Abbreviation: sh, small hairpin.

### 2.4 Reverse transcription‐quantitative PCR (RT‐qPCR)

Total RNA was isolated from the cultured cells and tissues using TriPure Isolation kit (BioTeke) and quantified by the NanoDrop 2000 UV photometer (Thermo Fisher Scientific, Inc.). The RNA was reverse‐transcribed into cDNA using a reverse transcription kit (No. 036a, Takara). PCR was performed using a PCR kit (item No. 820, Takara) with the following cycling parameters: 94°C for 5 mins, and 45 cycles of 94°C for 55 s, 60°C for 25 s and 72°C for 30 s. U6 and GAPDH were, respectively, used as the internal controls for miRNAs and other RNAs. The primer sequences are listed in Table 1.

### 2.5 Western blot analysis

The RIPA lysis buffer containing protease inhibitors (# KGP250, KeyGEN BioTECH, Nanjing, China) was used to extract PCa cell protein following the standard protocol. Then, equal amounts of proteins in the cell lysates were separated by SDS/PAGE gels (4%–12%, Bio‐Rad) and electronically transferred onto polyvinylidene fluoride (PVDF, Millipore) membranes. The membranes were then blocked with 5% bovine serum albumin (BSA) and incubated overnight at 4°C with the following specific primary antibodies: N Cadherin Antibody (T55015, Abmart), Vimentin Antibody (T55134, Abmart), GAPDH Antibody (MA9166, Abmart), IL10 Antibody (TD6894, Abmart), p62/SQSTM1 Antibody (T55546, Abmart), Beclin 1 Antibody (T55092, Abmart), LC3A/B Antibody (TA5402, Abmart), STAT3 Antibody (T55292, Abmart), Phospho‐STAT3 (Y705) Antibody (T56566, Abmart), JAK2 Antibody (T55287, Abmart) and Phospho‐JAK2 (Y1007 + Y1008) Antibody (T56570, Abmart). Subsequently, horseradish peroxidase (HRP)‐conjugated secondary antibody was used to incubate the samples for 1 h at room temperature. The bands were visualized using the enhanced chemiluminescence (ECL) detection system (Pierce Biotechnology, Rockford, IL, United States).

### 2.6 EdU assay

The proliferation rate of BLCA cells in vitro was determined by evaluating EdU incorporation with an EdU assay kit (C0071S, Beyotime) according to the manufacturer's instructions. Briefly, the cells were fixed and permeabilized, labelled with 5‐ethyl‐2 ‘‐deoxyuridine (EdU), counterstained with DAPI and observed under a fluorescence microscope.

### 2.7 Wound healing assay

The transfected cells were seeded in 6‐well plates and cultured till 100% confluent. The monolayer was scratched with a sterile 10 μL needle tip and washed with PBS to remove the dislodged cells. Images of the wound region were taken at 0 and 24 h, and migration rate was calculated as (migration distance/scratch width) × 100%.

### 2.8 Flank xenograft tumour model

The animal experiments were approved by the China Medical University Animal Welfare Laboratory and Ethics Committee (approval number: KT2020037) and conducted in accordance with the guidelines of general international laboratory.^43^ Twelve 6‐week‐old male BALB/c mice were purchased from Huafukang Biotech and housed under pathogen‐free ambient conditions (24 ± 1°C, 45%–55% humidity, 12 h light/dark) with ad libitum access to food and water. The mice were randomly divided into the circ‐NC and shRNA‐1 groups (*n* = 6 each) and injected with the suitably transfected UM‐UC‐3 cells into the flank regions (100 uL; 1 × 10^6^ cells/mouse). The tumours were measured every 3 days, and the volume was calculated as (length × width^2^)/2. The mice were euthanized after anaesthesia at 3 weeks after injection, and the maximum diameter of tumour was measured. All mice were administered vaporized isoflurane (inhaled) for anaesthesia at a concentration of 2.0% for induction and 1.0% for maintenance. Afterwards, the mice were sacrificed by rapid cervical dislocation. The mice with relaxed muscles were judged as dead when no breathing and no nerve reflex were observed. The xenograft tumours were harvested for further study.

### 2.9 Enzyme‐linked immunosorbent assay (ELISA)

IL‐10 levels in the culture supernatants were measured using an ELISA kit (Human IL‐10 enzyme‐linked immunosorbent Serological assay kit, Beyotime) according to the instructions. The absorbance was measured at 570 nm with a spectrophotometer, and IL‐10 concentration was determined using a standard curve as a reference.

### 2.10 BLCA patient samples

For circRNA expression analysis, we obtained 20 pathologically diagnosed BLCA tumour samples from the Second Affiliated Hospital of Xi'an Medical University. Tumour samples were collected continuously throughout 2021. Among them, 10 tumours were in situ tumours at T1 stage. Although bladder carcinoma in situ is classified as stage 1, complete bladder resection will be performed due to its potential for muscle invasion and metastasis.^44^ Meanwhile, the para‐cancerous normal tissues of the remaining few T1 stage patients are obtained from 8‐point random biopsies. In the remaining small number of T1 stage patients, para‐normal tissue was derived from an 8‐point random biopsy. The remaining tumour samples were all in situ tumours at or above the T2 stage, and the samples were tumour tissues and corresponding adjacent non‐cancerous bladder tissues. Corresponding adjacent non‐cancerous tissues were obtained ≥5 mm from the tumour site. At regular follow‐up, disease‐free‐survival (DFS) was identified from the date of surgery to the first evidence of clinical recurrence. The samples used in this study were approved by the Medical Ethics Committee of the Second Affiliated Hospital of Xi'an Medical University (approval number: X2Y202112L) and were used with the informed consent of each patient.

### 2.11 TAMs co‐culture and migration assays

The monocytes were induced with 100 ng/mL phorbol‐12‐myristate‐13‐acetate (PMA) for 24 h to obtain M0 TAMs (Beyotime, Shanghai, China). The TAMs were induced to the M2 phenotype with 20 ng/mL IL‐13 and IL‐4 (AF‐200‐04, AF‐200‐13, PeproTech) for 48 h. To assess the migratory capacity of TAMs, migration experiments were conducted using a transwell chamber with a pore size of 5.0 μm. The treated BLCA cells were cultured in a 24‐well plate, while M2 TAMs were introduced into the transwell chamber. In order to investigate the impact of IL‐10, TAMs were subjected to treatment with rh‐IL‐10 (HY‐P7030A, MedChemExpress) prior to the commencement of the experiment. Following a 48‐h treatment period, a transwell migration assay was conducted using crystal violet staining. In the co‐culture setting, circLOC729852‐overexpressed or knockdown BLCA cells were introduced into the upper layer of 5.0 μm transwell cells, while TAMs were inoculated into 24‐well plates. After 48 h of co‐culture, the macrophages present in the 24‐well plate were collected for subsequent experimental procedures. To identify macrophage surface markers, cells were stained with fluorophore‐conjugated antibodies against BSG/CD147 Rabbit Antibody (T40034, Abmart), Anti‐CD68 antibody (ab31630, Abcam), Anti‐CD80 antibody (ab134120, Abcam), Anti‐CD86 antibody (ab239075, Abcam), Mannose Receptor (MRC1) Rabbit Antibody (T40027, Abmart) and Goat Anti‐Rabbit lgG AF 594 (M21014, Abmart). Subsequently, flow cytometry analysis was conducted using a FACSCalibur flow cytometer (BD Biosciences) to quantify the stained cells.

### 2.12 RNA pulldown assay

To pull down the miRNA by circRNA, F2‐sense probe was synthesized, and F2‐antisense probe was used as a control. CircRNA pulldown assay was carried out using F2‐RNA pulldown kit (FI8701‐12 T). All procedures followed the manufacturer's instructions. Then, the final RNA was extracted by TRIzol (Invitrogen, United States) and analysed by RT qPCR.

### 2.13 Immunohistochemistry (IHC)

For IHC, the xenograft tissue paraffin sections were incubated with antibodies against IL‐10 (TD6894, Abmart), CD14 (T55722, Abmart) and CD208 (PA5969, Abmart). In addition, we also detected the expression of IL‐10, CD14 and CD208. Images were observed under an Olympus multifunction microscope (Olympus BX51, Tokyo, Japan). All evaluations were performed by three independent senior pathologists using the same microscope.

### 2.14 Statistical analysis

GraphPad Prism 9.5.1 was used for all statistical analyses. The differences between the two groups were analysed using the unpaired Student test, and three or more groups were compared by one‐way ANOVA. *p*‐value <0.05 was considered statistically significant.

## 3 RESULTS

### 3.1 CircLOC729852 is upregulated in BLCA and portends poor prognosis

**FIGURE 1:**
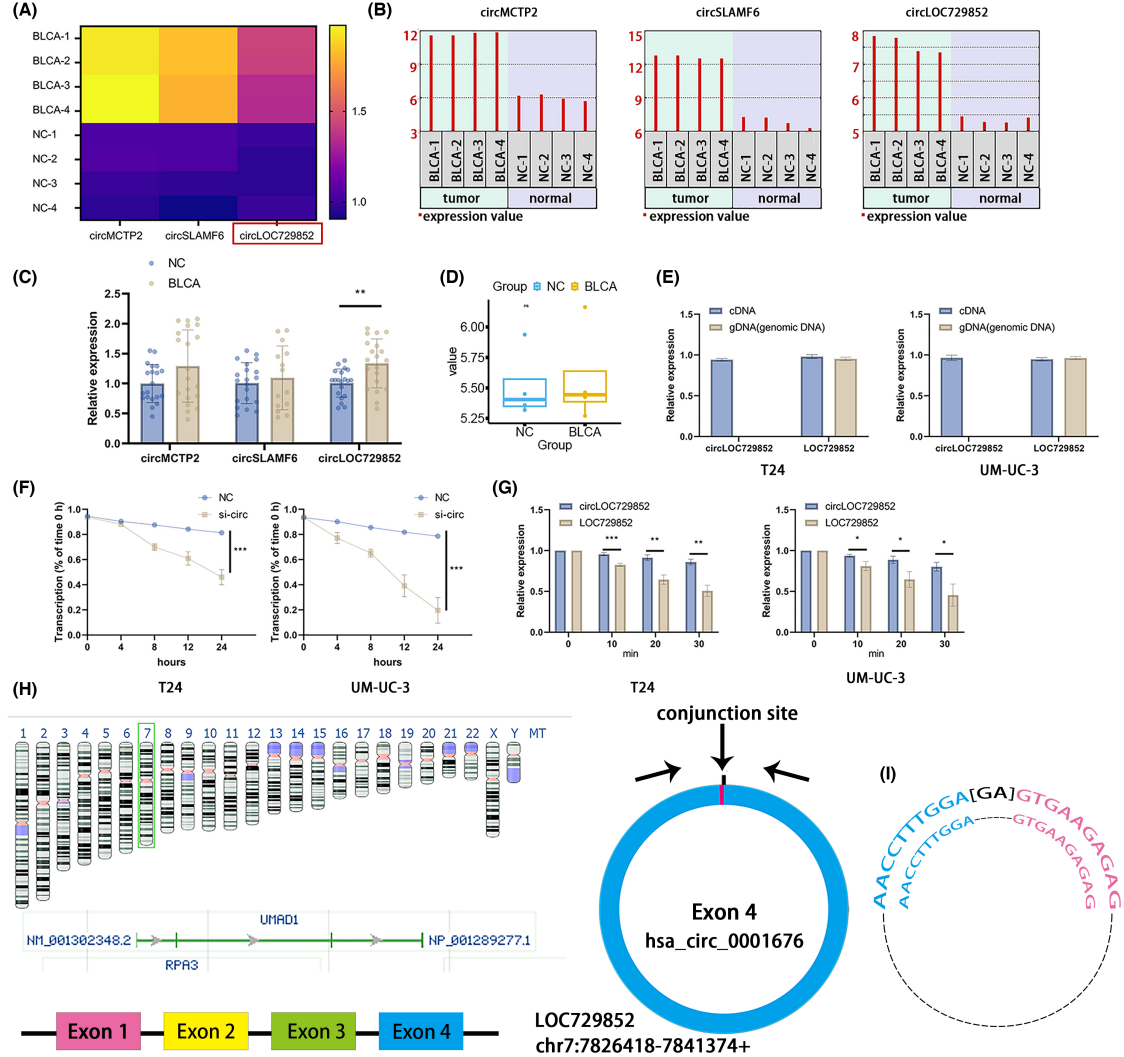
CircLOC729852 is overexpressed in BLCA. (A) Relative expression heat map of circMCTP2, circSLAMF6 and circLOC729852 in BLCA in GSE92675 dataset. (B) Expression levels of circMCTP2, circSLAMF6 and circLOC729852 in BLCA tissues and paracancer tissues according to GSE92675 dataset. (C) Expression levels of circMCTP2, circSLAMF6 and circLOC729852 in 20 paired BLCA and normal tissues. (D) Expression levels of circLOC729852 in BLCA tissues and paracancer tissues according to GSE147984 dataset. (E) Amplification results of circLOC729852 and its host gene LOC729852 in cDNA and gDNA. (F) RNase R assay results of circLOC729852 and its host gene LOC729852. (G) Results of actinomycin D assay for circLOC729852 and its host gene LOC729852. (H) circLOC729852 is formed by its host gene LOC729852. (I) Sanger sequencing results of the Rqt‐PCR amplification product of circLOC729852 and base pairing diagram of the circLOC729852 primer.

We identified 15 differentially expressed circRNAs in the GSE92675 dataset, of which circMCTP2, circSLAMF6 and circLOC729852 (also known as UMAD1) were matched to host genes. The heatmap of these circRNAs in BLCA tissues is shown in Figure 1A, and their relative expression levels in the three pairs of NC and BLCA tissues have been summarized in Figure 1B. We also analysed the expression of these candidate circRNAs in 20 paired BLCA and normal bladder tissue specimens and found that circLOC729852 was significantly upregulated in the BLCA samples (Figure 1C). To verify the ring structure of circLOC729852, we amplified the LOC729852 gene and circRNA in the genomic DNA (gDNA) and cDNA from BLCA cell lines. In addition, we explored the expression of circLOC729852 in NC and BLCA in the GSE147984 dataset, and the results showed that circLOC729852 was highly expressed in BLCA (Figure 1D). While LOC729852 could be amplified in both cDNA and gDNA, circRNA could be only amplified in cDNA (Figure 1E). In addition, Actinomycin D and RNase R assays also demonstrated the higher stability of circLOC729852 (Figure 1F, G). LOC729852 is located on chromosome 7 and has four exons, and circLOC729852 is transcribed from the fourth exon (Figure 1H). RT‐qPCR was performed on circLOC729852, and the products were sequenced; the ring structure was confirmed by sequencing (Figure 1I). These results suggested that circLOC729852 is highly expressed in BLCA and is formed by post‐transcriptional splicing of LOC729852.

**FIGURE 2:**
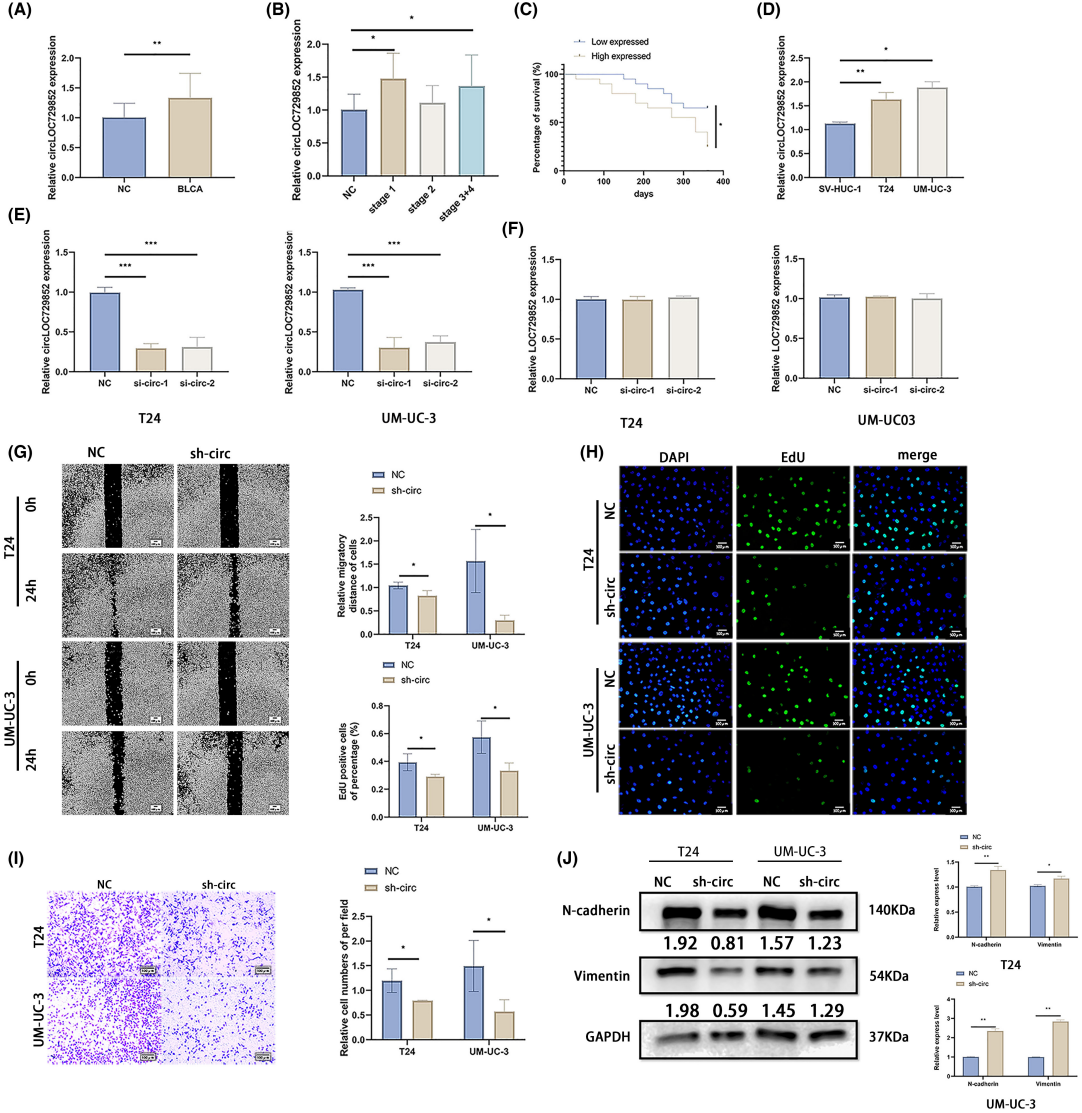
CircLOC729852 correlates with the clinicopathological features of BLCA. (A) CircLOC729852 levels in BLCA and adjacent normal tissues. (B) Relationship between circLOC729852 level and BLCA clinical pathological stages. (C) Relationship between circLOC729852 level and prognosis of BLCA patients. (D) CircLOC729852 expression in SV‐HUC‐1 T24, and UM‐UC‐3 cells. (E) CircLOC729852 level in BLCA cells after transfection with NC, sh‐circ‐1 and sh‐circ‐2. (F) The host gene LOC729852 level after transfection with NC, sh‐circ‐1 and sh‐circ‐2. (G) After transfection with NC and sh‐circ, the BLCA cells' migration was examined by wound healing assay. (H) After transfection with NC and sh‐circ, the BLCA cells' proliferation was examined by EdU assay. (I) After transfection with NC and sh‐circ, the BLCA cells' migration was examined by transwell migration assay. (J) After transfection with NC and sh‐circ, Western blotting assay was used to examine the EMT.

To further explore the role of circLOC729852 in BLCA, we analysed its expression in 20 paired BLCA and para‐tumour (>5 cm delineation) tissues, and its correlation to clinicopathological parameters. As shown in Figure 2A, circLOC729852 was highly expressed in the BLCA tissues (Figure 2A). The clinical characteristics of the 20 patients are summarized in Table 2. CircLOC729852 was upregulated in stage 1 and stage 3/4 tumours (Figure 2B). As shown in the Figure 2C, circLOC729852 was associated with poor prognosis in BLCA patients.

**TABLE 2:** Clinicopathological features of bladder cancer patients.

Characteristics	Number of cases
Mean age (range) (years)	68 (39–86)
Sex	
Male/Female	17/3
Histological type	
Urothelial carcinoma/Squamous cell	19/1
Pathology T stage	
1/2/3/4	10/4/2/4

### 3.2 CircLOC729852 accelerated migration and proliferation capacity of BLCA cells in vitro

CircLOC729852 was highly expressed in BLCA cell lines (T24, UM‐UC‐3) compared to normal bladder tissue cells (SV‐HUC‐1) (Figure 2D). We designed two shRNAs targeting circLOC729852, of which sh‐circ‐1 showed higher knockdown efficiency in the BLCA cell lines (Figure 2E). Thus, sh‐circ‐1 was used for the subsequent experiments. As shown in Figure 2F, neither shRNA had any significant effect on the host gene LOC729852. Knocking down circLOC729852 inhibited the migration of BLCA cells in the scratch healing and transwell assays (Figure 2G, I). Furthermore, BLCA cells with circLOC729852 knockdown showed lower proliferation rate in the EdU assay (Figure 2H). We also analysed the expression of epithelial mesenchymal transition (EMT) markers in the control and circLOC729852‐knockdown cells, and found that N‐cadherin and Vimentin were downregulated in the latter (Figure 2J). Taken together, these results suggest that circLOC729852 acts as an oncogene in BLCA, and promotes the proliferation, migration and EMT of the tumour cells.

### 3.3 CircLOC729852 directly binds to and inhibits miR‐769‐5p

**FIGURE 3:**
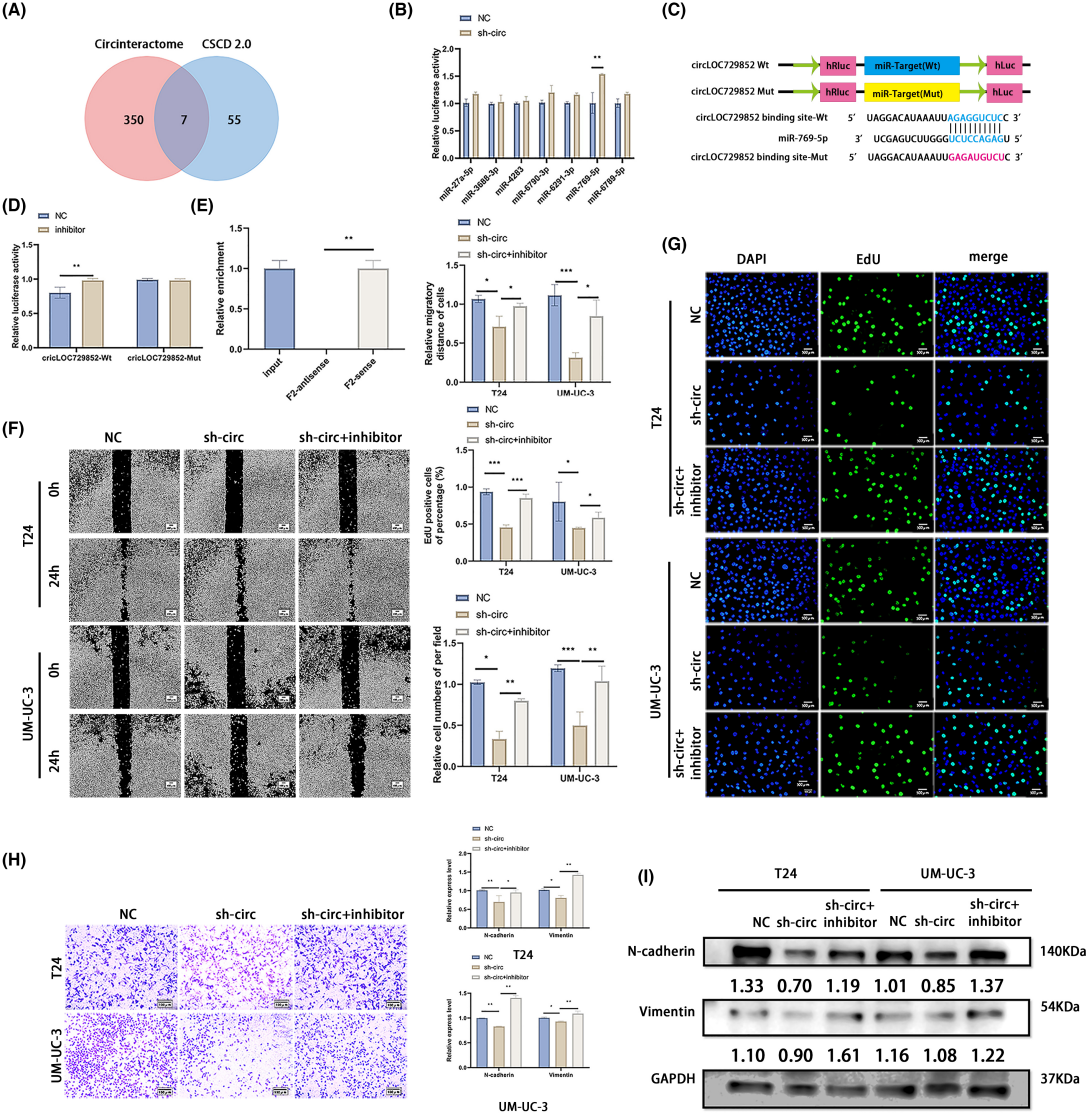
CircLOC729852 facilitates proliferation and migration of BLCA cells. (A) Circinteractome and CSCD 2.0 databases were performed to predict the target miRNA of circLOC729852, and the results were intersected. (B) Expression of the seven candidate targets of circLOC729852, miR‐27a‐5p, miR‐3688‐3p, miR‐4283, miR‐6790‐3p, miR‐6821‐3p, miR‐769‐5p and miR‐6789‐5p after transfection of sh‐circ and NC. (C) CircLOC729852 luciferase reporter vector with a mutant (Mut) or wild‐type (Wt) miR‐769‐5p binding site. (D) The effect of miR‐769‐5p on circLOC729852 luciferase activity was demonstrated by dual luciferase assay. (E) The RNA pulldown assay performed in UM‐UC‐3 cells using circLOC729852 F2‐sense and F2‐antisense probes. (F) After transfection with NC, sh‐circ and sh‐circ+inhibitor, rescue experiment was performed, the BLCA cells' migration was examined by wound healing assay. (G) After transfection with NC, sh‐circ and sh‐circ+inhibitor, rescue experiment was performed, and the BLCA cells' proliferation was examined by EdU assay. (H) Rescue experiment was performed after transfection with NC, sh‐circ and sh‐circ+inhibitor, and transwell migration assay was used to examine the migration ability of BLCA cells. (I) After transfection with NC, sh‐circ and sh‐circ+inhibitor, rescue experiment was performed, and Western blotting assay was used to examine the EMT.

We screened for the putative downstream target miRNAs of circLOC729852 using the circinteractome and CSCD2.0 databases, and, respectively, obtained 390 and 63 miRNAs. As shown in Figure 3A, miR‐27a‐5p, miR‐3688‐3p, miR‐4283, miR‐6790‐3p, miR‐6821‐3p, miR‐769‐5p and miR‐6789‐5p were common to both databases. The expression levels of these miRNAs were analysed in the circLOC729852‐knockdown cells, and only miR‐769‐5p was significantly upregulated (Figure 3B). To confirm the interaction between circLOC729852 and miR‐769‐5p, we constructed dual luciferase reporter plasmids with mutant and wild‐type miR‐769‐5p binding sites of circLOC729852 (Figure 3C), and co‐transfected the respective reporter plasmids with miR‐769‐5p or the empty vector into HEK‐293 T cells. As shown in Figure 3D, miR‐769‐5p significantly decreased the luciferase activity of the wild‐type but not the mutant circLOC729852 reporter construct (Figure 3D). These results indicated that circLOC729852 can directly bind to miR‐769‐5p.

### 3.4 MiR‐769‐5p inhibition rescued BLCA cells from the effects of circLOC729852 knockdown

Subsequently, RNA pulldown experiments were performed using F2‐sense and F2‐antisense probes. The results demonstrated that miR‐769‐5p was substantially pulled down by the F2‐sense probe rather than the F2‐antisense in UM‐UC‐3 cells with circLOC729852 overexpression, suggesting that circLOC729852 might directly binds to miR‐769‐5p (Figure 3E). To determine whether the oncogenic effects of circLOC729852 are mediated via miR‐769‐5p, we co‐transfected BLCA cells with miR‐769‐5p inhibitor and sh‐circLOC729852, and evaluated their migration and proliferation. The inhibitory effect of circLOC729852 knockdown on the migration ability of BLCA cells was reversed by blocking miR‐769‐5p (Figure 3F, H). Likewise, miR‐769‐5p inhibition also restored the proliferative capacity of the BLCA cells with circLOC729852 knockdown (Figure 3G). Finally, inhibition of miR‐769‐5p also upregulated the N‐cadherin and Vimentin in the circLOC729852‐knockdown cells (Figure 3I). Taken together, miR‐769‐5p inhibition rescued the proliferation, migration and EMT of circLOC729852‐knockdown cells, indicating that circLOC729852 acts as a sponge for miR‐769‐5p and its oncogenic function in BLCA depends on the suppression of miR‐769‐5p.

### 3.5 CircLOC729852 regulated autophagy in the BLCA cells via the miR‐769‐5p/IL‐10 axis

**FIGURE 4:**
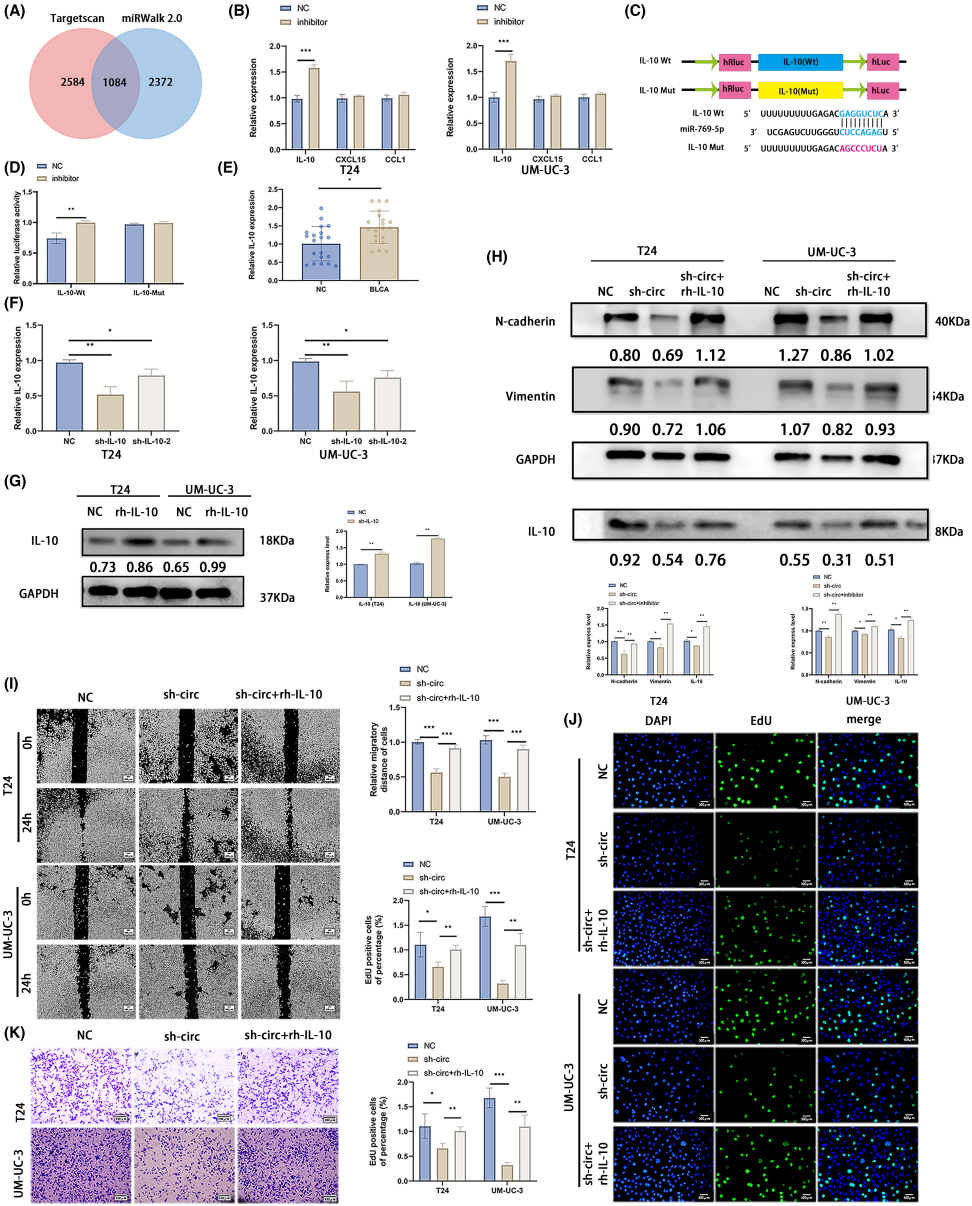
CircLOC729852 exerts its oncogenic effects by suppressing miR‐769‐5p. (A) The target genes of miR‐769‐5p were predicted by targetscan and miRwalk 2.0 databases, and the intersection was obtained. (B) Expression levels of three candidate genes of miR‐769‐5p, IL‐10, CXCL15 and CCL1, after transfection with NC and inhibitor of BLCA. (C) Illustration of IL‐10 Wt and Mut luciferase reporter vectors. (D) The relative activity of luciferase in 293 T cells co‐transfected with IL‐10‐Mut or IL‐10‐Wt and miR‐769‐5p NC or inhibitor was measured by dual luciferase reporter method. (E) IL‐10 level in 20 paired BLCA and adjacent normal tissues. (F) IL‐10 level in BLCA cells transfected with NC, sh‐IL‐10 and Sh‐IL‐10‐2. (G) IL‐10 levels in BLCA cells after transfection with NC and rh‐IL‐10. (H) After transfection with NC, sh‐circ and sh‐circ+inhibitor, the EMT marker and IL‐10 levels in BLCA cells were detected by Western blotting. (I) After transfection with NC, sh‐circ and sh‐circ+rh‐IL‐10, rescue experiment was performed, and the BLCA cells' migration was examined by wound healing experiment. (J) After transfection with NC, sh‐circ and sh‐circ+rh‐IL‐10, rescue experiment was performed, and the BLCA cells' proliferation was examined by EdU assay. (K) After transfection with NC, sh‐circ and sh‐circ+rh‐IL‐10, rescue experiments were performed, and transwell migration assay was performed to examine the BLCA cells' migration.

The potential target genes of miR‐769‐5p were predicted from the Targetscan and MiRwalk2.0 databases, which, respectively, identified 3669 and 7164 target genes, of which 1084 were common to both (Figure 4A). The protein interaction network (PPI) of these genes was mapped, and three immune‐related hub genes, including IL‐10, CXCL15 and CCL1, were identified. Furthermore, knocking down miR‐769‐5p in the BLCA cells led to a significant increase in IL‐10 levels but did not affect the other hub genes (Figure 4B). Dual luciferase reporter assay was performed to confirm the interaction between IL‐10 and miR‐769‐5p. HEK‐293 T cells were co‐transfected with reporter plasmid containing mutant or wild‐type miR‐769‐5p binding sites of IL‐10 (Figure 4C) and miR‐769‐5p or the empty vector. As shown in Figure 4D, miR‐769‐5p significantly decreased the luciferase activity of the wild‐type IL‐10 reporter construct, indicating that miR‐769‐5p directly targets IL‐10.

**FIGURE 5:**
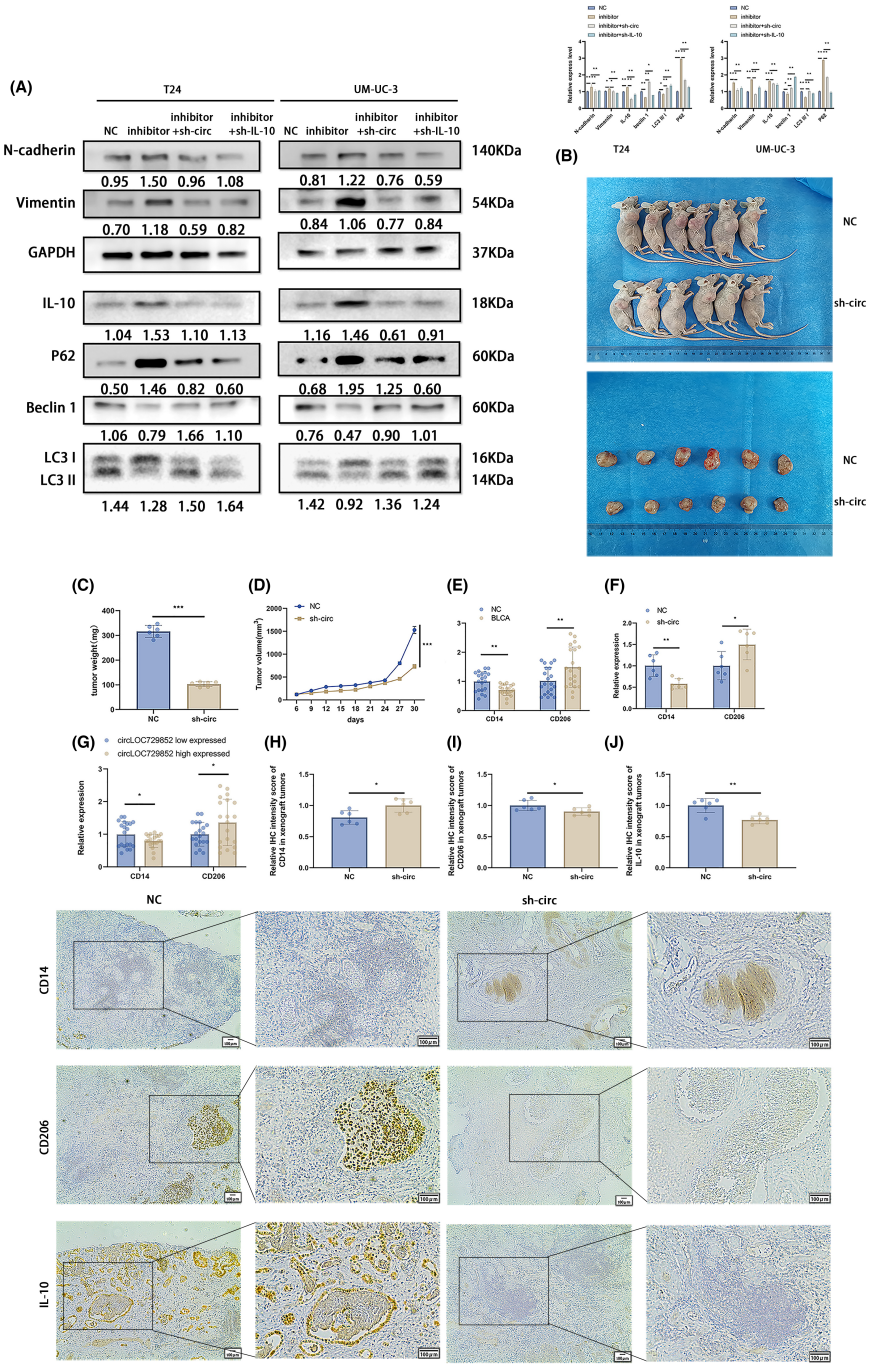
IL‐10 is a direct target of miR‐769‐5p and regulates the autophagy pathway via the circLOC729852/miR‐769‐5p/IL‐10 axis. (A) Transfection of NC, inhibitor, inhibitor+sh‐circ and inhibitor+sh‐IL‐10, rescue experiment was performed to examine the expression of autophagy, EMT marker and IL‐10 protein. (B) UM‐UC‐3 cells after transfection of NC and sh‐circ were used to conduct Xenograft tumour model in nude mice. (C) Tumour weight in NC group and sh‐circ transfected mice. (D) Every 3 days, during the process of in vitro tumour formation, the tumour volume was measured and calculated, and the volume growth curve was obtained. (E) Expression of CD14 and CD206 in 20 paired BLCA and adjacent normal tissues. (F) Expression of CD14 and CD206 in 6 paired mouse tumours. (G) The expression of CD14 and CD206 in circLOC729852 high and low expressed tissues. (H) IHC detected the infiltration of CD14+ macrophages in xenograft tumours. (I) IHC detected the infiltration of CD206+ macrophages in xenograft tumours. (J) IHC detection of IL‐10 expression in xenograft tumours.

Examination of the 20 paired normal bladder and BLCA tissues further revealed that IL‐10 was upregulated in the BLCA tissues (Figure 4E). Furthermore, circLOC729852 knockdown in BLCA cells significantly reduced IL‐10 expression, which was reversed by miR‐769‐5p inhibition (Figure 4H), indicating that IL‐10 is directly downstream of miR‐769‐5p. We designed two shRNAs targeting IL‐10, of which sh‐IL‐10‐1 showed higher knockdown efficiency in the BLCA cell lines (Figure 4F). Thus, sh‐IL‐10‐1 was used for the subsequent experiments. In addition, IL‐10 human recombinant protein (rh‐IL‐10) upregulated the IL‐10 expression (Figure 4G). N‐cadherin and Vimentin were downregulated in circLOC729852‐knockdown cells, which was restored by rh‐IL‐10 (Figure 4H). Knocking down circLOC729852 inhibited the migration of BLCA cells in the scratch healing and transwell assays, which was restored by rh‐IL‐10 (Figure 4I, J). Furthermore, BLCA cells with circLOC729852 knockdown showed lower proliferation rate in the EdU assay, which was restored by rh‐IL‐10 (Figure 4J). Inhibition of miR‐769‐5p upregulated the levels of N‐cadherin and Vimentin. The effect of inhibitor on EMT was abrogated by sh‐circ and rh‐IL‐10. Inhibition of miR‐769‐5p upregulated the level of P62 and decreased the levels of Beclin 1 and the rate of LC3 II/I, and the acceleration effect of inhibitor in autography was abrogated by sh‐circ and rh‐IL‐10 (Figure 5A). Taken together, these results illustrate that circLOC729852 inhibits the autophagy pathway in BLCA cells by suppressing miR‐769‐5p and upregulates IL‐10, thereby promoting tumour progression.

### 3.6 CircLOC729852 promoted BLCA growth and M2 polarization of TAMs in vivo

To investigate the function of circLOC729852 in vivo, we established xenografts of control and sh‐circ UM‐UC‐3 cells in nude mice (Figure 5B). The tumours were measured every 3 days and weighed at the end of the experiment. The tumours derived from the sh‐circ cells were lighter (Figure 5C) and smaller compared to that in the control group and also showed slower growth (Figure 5D). These results suggest that circLOC729852 promotes BLCA progression in vivo.

IL‐10 is secreted by multiple cells, especially M2 TAMs, and regulates cell growth, differentiation, inflammatory and immune responses, and promote cancer progression.^45^, ^46^ In addition, BLCA tissues show higher abundance of M2 TAMs compared to the para‐cancerous tissues.^47^, ^48^ Consistent with this, the monocyte marker CD14 was expressed at low levels in the BLCA tissues relative to the paired normal tissues, while CD206 expression was high in the tumours (Figure 5E). Furthermore, the xenograft tissues of mice inoculated with the circLOC729852‐knockdown BLCA cells showed increased expression of CD14 and a decrease in CD206 expression compared to that of the control group (Figure 5F), indicating lower abundance of the M2 TAMs. Interestingly, the number of TAMs (CD206+) in BLCA patients' tissues with circLOC729852 upregulation was significantly higher than that in tissues with circLOC729852 downregulation, while monocyte (CD14+) in tissues with circLOC729852 upregulation was significantly lower than that in tissues with circLOC729852 downregulation (Figure 5G). Furthermore, we obtained consistent results in BALB/c mice xenograft tumours. The number of TAMs (CD206+) in xenograft tumours was significantly decreased in the mice sh‐circ group compared to the NC group, and the number of monocyte (CD14+) in xenograft tumours was significantly increased in the mice sh‐circ group compared to the NC group (Figure 5H, I). We further found that IL‐10 in xenograft tumours was downregulated in in the mice sh‐circ group compared to the NC group via IHC assay (Figure 5J). These results suggested that the expression of circ LOC729852 is positively correlated with M2 macrophage infiltration, and circ LOC729852 may induce M2 macrophage colonization. Based on these results, we can surmise that circLOC729852 may induce M2 polarization of the TAMs.

### 3.7 CircLOC729852 polarized TAMs to the M2 phenotype by increasing IL‐10 production via activation of the JAK2/STAT3 signalling pathway

**FIGURE 6:**
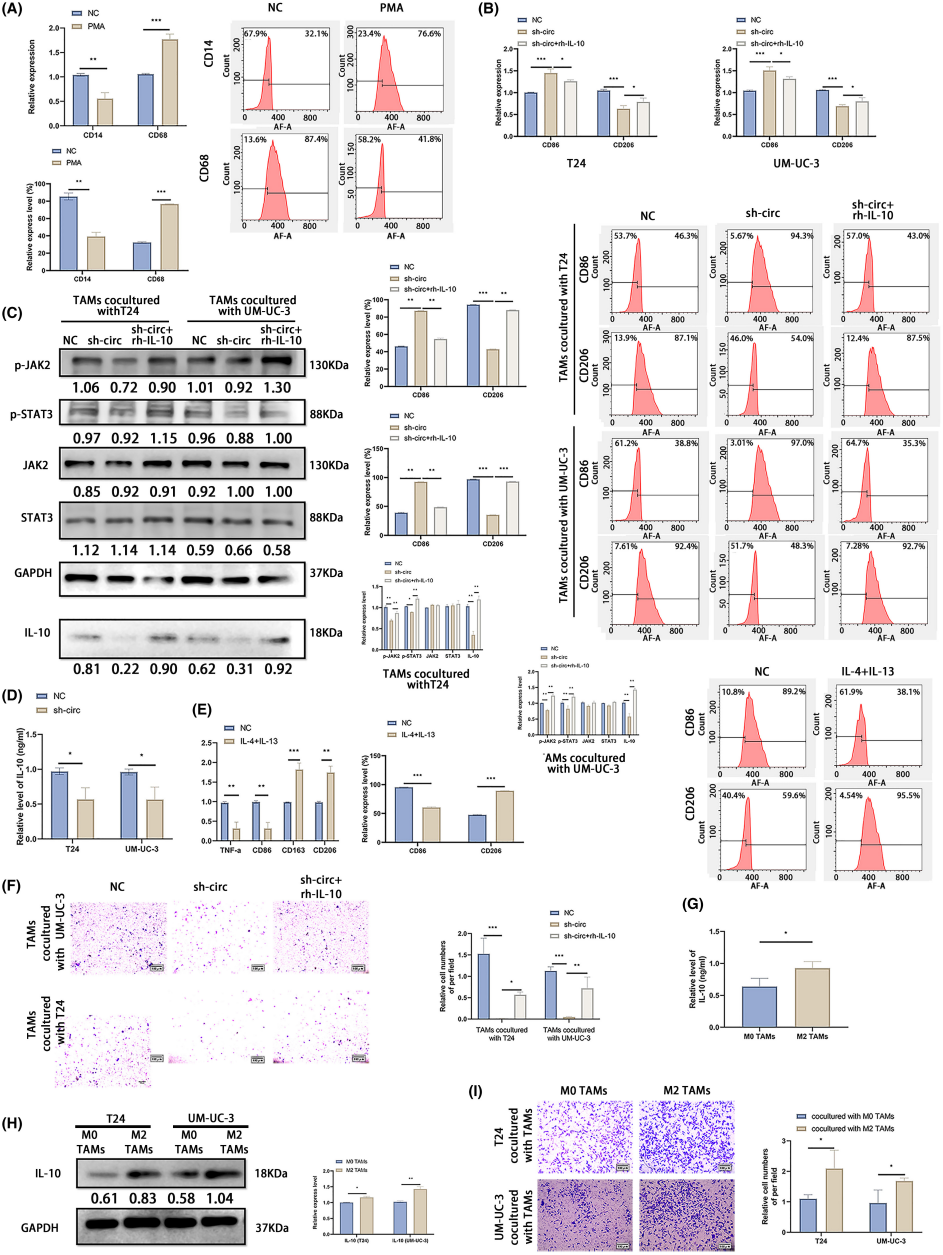
CircLOC729852 promotes M2 macrophage polarization and recruitment via IL‐10. (A) The percentage of CD14 and CD68 positive cells was examined by RT‐qPCR and flow cytometry. (B) The percentage of CD86 and CD206 positive cells was examined by RT‐qPCR and flow cytometry. (C) M0 TAMs co‐cultured with BLCA cells transfected with NC, sh‐circ and sh‐circ+rh‐IL‐10 were performed rescue experiments, and western blotting was used to detect JAK2/STAT3 signalling pathway and IL‐10 expression level. (D) IL‐10 secretion levels in the supernatant of BLCA medium after transfection with NC and sh‐circ. (E) The percentage of CD86, TNF‐a, CD206 and CD163 positive cells was determined by RT‐qPCR and the percentage of CD86 and CD206 positive cells was examined by flow cytometry. The expression of CD86, TNF‐a, CD206 and CD163 in M0 TAMs cells induced by IL‐13 and IL‐4. (F) Rescue experiments were performed on M2‐Tams co‐cultured with BLCA cells transfected with NC, sh‐circ and sh‐circ +rh‐IL‐10, and the transwell assay was used to examine the migration of M2 TAMs. (G) IL‐10 secretion levels in the medium of M0 and M2 TAMs. (H) IL‐10 expression in BLCA cells co‐cultured with M0, M2 TAMs. (I) Migration ability of BLCA cells co‐cultured with M0, M2 TAMs.

To explore the effect of circLOC729852 on macrophage polarization, we induced the differentiation of THP‐1 monocytes to the CD86+ M0 macrophages with 100 mg/mL PMA (Figure 6A) and co‐cultured them with circLOC729852‐knockdown BLCA cells in the presence or absence of rh‐IL‐10. As shown in Figure 6B, CD86 was downregulated and CD206 was upregulated in the co‐cultured macrophages. In addition, circLOC729852 knockdown also decreased the percentage of CD206+ M2 macrophages and increased that of the CD86+ M1 macrophages (Figure 6B). However, rh‐IL‐10 reversed the effects of circLOC729852 knockdown. Thus, circLOC729852 likely induces M2 polarization of TAMs through IL‐10. M2 polarization is mediated by the JAK2/STAT3 pathway.^49^, ^50^, ^51^, ^52^, ^53^ CircLOC729852 knockdown significantly reduced the levels of phosphorylated STAT3 and JAK2 in the co‐cultured macrophages, but had no effect on total STAT3 and JAK2 expression levels. The inhibitory effect of sh‐circ on the JAK2/STAT3 signalling pathway was abrogated by rh‐IL‐10 (Figure 6C). In addition, TAMs co‐cultured with circLOC729852‐knockdown BLCA cells expressed lower levels of IL‐10, which was counteracted by rh‐IL‐10 (Figure 6C). Furthermore, sh‐circ also reduced IL‐10 levels in the culture supernatant of BLCA cells (Figure 6D). To determine the effect of circLOC729852 on the migration of M2 TAMs, we induced M2 polarization in vitro using IL‐13 and IL‐4. As shown in Figure 6E, the cytokines reduced the expression of the M1 markers TNF‐a and CD86, while significantly increasing that of the M2 markers CD163 and CD206. In addition, IL‐4 and IL‐13 also increased the percentage of CD86+ M2 macrophages and decreased that of the CD206+ M1 macrophages (Figure 6E). The M2 macrophages were co‐cultured with BLCA cells as described, and as shown in Figure 6F, circLOC729852 knockdown significantly decreased the migration of the M2 macrophages in the transwell assay, which was restored by rh‐IL‐10. These results suggested that circLOC729852 promote the recruitment of M2 macrophages to the tumours by upregulating IL‐10. The M2 TAMs characteristically secrete IL‐10,^45^, ^46^ and consistent with this, IL‐10 levels were high in the culture medium of the induced M2 macrophages (Figure 6G). Furthermore, BLCA cells co‐cultured with the M2 TAMs not only expressed higher levels of IL‐10 (Figure 6H) but also showed increased migration capacity (Figure 6I). Thus, M2 TAMS may facilitate BLCA progression by secreting IL‐10 in the TME and promoting tumour cell migration.

## 4 DISCUSSION

BLCA is associated with high mortality and morbidity rates, resulting in considerable socio‐economic burden.^54^, ^55^ In recent years, the molecular mechanisms underlying BCLA tumorigenesis have been increasingly elucidated, and several BLCA‐specific biomarkers have been identified. CircRNAs are noncoding RNAs that are aberrantly expressed in multiple cancers, including BLCA.^56^, ^57^, ^58^, ^59^, ^60^ We screened the GEO database for aberrantly expressed circRNAs in BLCA and found that circLOC729852 is highly expressed in the BLCA tissues compared to normal bladder tissues, and its expression level correlates positively with the tumour stage. In vitro and in vivo functional assays confirmed that circLOC729852 functions as an oncogene in BLCA and promotes tumour progression by inducing the recruitment and M2 polarization of TAMs.

It has been reported that exogenous addition of IL‐10 directly to gastric cancer cells can promote the proliferation of gastric cancer cells.^61^ This is due to the activation of a c‐mesenchymal epithelial transition protein/STAT3 signalling pathway by IL‐10 to promote the malignant behaviour of gastric cancer cells.^62^ IL‐10, IL‐5 and cytotrophins can activate JAK. JAK in turn activates STAT to phosphorylate STAT. After phosphorylation, STAT enters the nucleus and binds to specific DNA elements to exert its biological function. STAT1 and STAT3 abnormal activation can promote cell differentiation, proliferation, inhibiting cell apoptosis, IL‐10 can combine JAK2 and activate JAK2 / STAT3 signalling pathway, which play a biological effect.^63^

In bladder cancer, PRDX6 promoted the proliferation and cell cycle of bladder cancer cells through JAK2/STAT3 signalling pathway.^64^ In addition, EZH2 is highly expressed in bladder cancer, while JAK2/STAT3 signalling pathway is abnormally activated. EZH2 can promote the proliferation and migration of bladder cancer cells by activating JAK2/STAT3 signalling pathway.^65^

Autophagy, a self‐engulfing programme, is an intracellular mechanism that degrades proteins and clears damaged organelles by melting media.^66^, ^67^ It has been reported that circRNA can affect tumour cells through autophagy.^68^ For example, circGSPT1, which is lowly expressed in gastric cancer, can encode GSPT1‐238aa protein, and the GSPase domain of GSPT1‐238aa protein can interact with the PI3K/Akt/mTOR signalling pathway. Class III PI3K in the pathway can regulate the formation of autophagic vesicles by forming a complex with Beclin 1, while AKT‐mediated phosphorylation of Beclin 1 can enhance the interaction of Beclin 1 with 14–3‐3 and vimentin proteins to form a complex, thereby regulating cell autophagy.^69^

Alterations in autophagy are associated with the carcinogenesis of cancers, of which BLCA is the first cancer to be associated with Beclin 1.^70^ At the same time, low expression of Beclin 1 is an independent indicator of poor prognosis of BLCA.^71^ Numerous evidences have shown that JAK2/SATA3 signalling pathway plays an important role in the regulation of autophagy.^72^, ^73^ Under normal circumstances, the activation of intracellular JAK2/STAT3 pathway is tightly controlled and the activation time is short. However, in the tumour microenvironment, due to the continuous action of chemical signals from cytokines, JAK activates STAT3 phosphorylation after activation, and p‐STAT3 continues to form a dimer into the nucleus to regulate the transcription of many autophagy‐related genes. Thus, it participates in the regulation of cell autophagy.^74^ Mcl‐1 is a protein associated with autophagy in the JAK2/SAT3 signalling pathway.^75^ MCL‐1 negatively regulates autophagy, mainly through the autophagy‐related gene Beclin 1.^76^, ^77^, ^78^ Beclin 1 is needed for the autophagy pathway is positive adjustment factor, Beclin 1 with type III PI3K/hVps34, forming complexes directly inducing autophagy. The MCL‐1 can be combined with Beclin 1 protein BH3 domain structure, thus inhibiting Beclin 1, autophagy and negative control cells.^76^, ^77^, ^78^ In addition, the agglomeration of P62 and autophagy is directly related to, such as TSPO can through direct action with P62, lead to agglomeration of P62 and inhibition of autophagy.^79^ In bladder cancer, CXCL12 upregulates the deubiquitinating enzyme CYLD through the JAK2/STAT3 signalling pathway, and CYLD inhibits autophagy in bladder cancer by inducing the accumulation of P62 through deubiquitination of P62.^80^ In addition, through the JAK2/STAT3 signalling pathway inhibitor Stattic also can inhibit the rise in JAK2/STAT3 signal pathway of P62,^80^ miR‐16‐5p can promote autophagy by upregulating Beclin 1 and downregulating P62, thus playing an inhibitory effect on bladder cancer.^81^

However, autophagy plays multiple roles in the occurrence and development of primary tumours.^82^ It can not only inhibit the occurrence and development of tumours, but also promote the metabolic adaptability of tumours to promote survival. Selective autophagy is a specific programme that mediates the degradation of cargoes for immune regulation or carcinogenesis.^83^ It is a more accurate analysis of the effect of autophagy, rather than the macroscopic perspective of inhibition or activation of autophagy.^83^ In addition, this study only detected the expression changes of autophagy‐related proteins at the protein level in cells, which still needs to be further verified by animal experiments and using more knowledge detection methods.

The TME consists of fibroblasts, endothelial cells and immune cells, which interact with the tumour cells and modulate cancer progression. Macrophages are the predominant immune cells in the TME and release cytokines that promote angiogenesis and tumour metastasis.^84^ TAMs polarize to the M2 and M1 phenotypes depending on the stimuli; while the M1 TAMs have immune‐boosting and anti‐tumour effects, M2 TAMs are immunosuppressive and promote tissue repair and tumour development. Both phenotypes co‐exist in all stages of tumour development, with the M1 type dominating in the early stages, and the M2 type in the middle and late stages. The polarization of the M1 TAMs to the M2 TAMs type promotes tumour progression.^85^

TAMs secrete large amounts of IL‐10,^45^, ^46^ which induces EMT of tumour cells through STAT3 activation and thus promotes metastasis.^86^, ^87^ In addition, IL‐10 also promotes M2 polarization through the JAK2/STAT3 pathway, which is involved in biological processes like differentiation, proliferation and immune regulation by cytokines.^49^ Studies show that TAMs tend to polarize to the M2 phenotype by activating the JAK2/STAT3 pathway. For instance, IL‐8, MAPK, IL‐6, IRF3, IL‐10 and even cigarette extracts can promote M2 polarization in TAMs by activating the JAK2/STAT3 pathway.^49^, ^50^, ^51^, ^52^, ^53^ On the other hand, ceramide and palmitic acid are known to inhibit M2 polarization by downregulating IL‐10.^88^ There is evidence that IL‐10 secreted by M2 TAMs facilitate colorectal cancer and breast cancer progression.^87^, ^88^

Increased infiltration of TAMs into the tumour tissues is often related to worse prognosis in multiple cancers.^89^, ^90^ Furthermore, TAM depletion has been shown to prevent tumour progression and metastasis, and re‐sensitize tumour cells to chemotherapy.^91^, ^92^ Inhibiting the recruitment of TAMs by blocking chemokines and receptors can also control tumour progression. The chemokine CCL5 promotes the recruitment and M2 polarization of TAMs upon binding to its specific receptor. Maraviroc (MVC), a small‐molecule CCR5 antagonist that prevents the entry of HIV into host cells, has also been used experimentally and clinically to inhibit TAMs recruitment and M2 polarization to improve cancer progression.^93^, ^94^, ^95^, ^96^, ^97^ Although IL‐10 levels in the TME cannot be reduced directly, the effect of IL‐10 can be weakened by specific inhibition of the IL‐10 receptor (IL‐10R), which consists of the IL‐10RA and IL‐10RB subunits. Several IL‐10R inhibitors have been reported that can block the binding of IL‐10 and IL‐10R, and potentially inhibit TAMs and cancer progression.^88^, ^98^

CircRNAs have been implicated in various cancers, including BLCA.^99^ For instance, knocking down has‐circRNA‐403,658 in BLCA cells accelerated apoptosis and inhibited tumour growth.^100^ In addition, overexpression of circMYLK facilitated angiogenesis and EMT in BLCA,^101^ while circ_0003221 knockdown suppressed the proliferation and motility of BLCA cells.^99^ Furthermore, several circRNAs have been identified that modulate the TME. CircCDR1 plays a key role in the infiltration of immune cells in tumour tissues, especially that of activated M2 TAMs, NK cells and CD8^+^ T cells.^102^ Likewise, circASAP1 mediates the infiltration of TAMs via the miR‐326/miR‐532‐5p‐CSF‐1 axis.^103^ Therefore, targeting TME via circRNAs is a viable therapeutic strategy for cancer. However, the exact molecular mechanisms through which circRNAs regulate TAMs are unclear. We found that circLOC729852 promotes the M2 polarization and infiltration of TAMs. The culture medium of BLCA cells with circLOC729852 knockdown promoted M2 TAMs recruitment and polarization via the circLOC729852/miR‐769‐5p/IL‐10 axis. CircLOC729852 upregulated IL‐10 expression in the BLCA cells via the miR‐769‐5p/IL‐10 axis, which in turn promoted the recruitment and M2 polarization in TAMs. In addition, the high levels of IL‐10 secreted from M2 TAMs may upregulate IL‐10 in BLCA cells and facilitate their migration.

To summarize, high circLOC729852 expression in BLCA promotes tumour progression and infiltration of M2 TAMs via the miR‐769‐5p/IL‐10 axis. Furthermore, the circLOC729852/miR‐769‐5p/IL‐10 axis is an attractive therapeutic target in M2 macrophage‐driven cancers.

## 5 CONCLUSION

**FIGURE 7:**
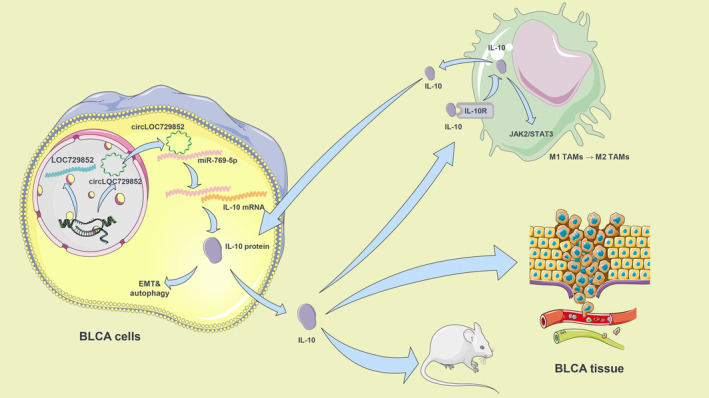
The schematic shows the mechanism by which circLOC729852 promotes BLCA progression.

CircLOC729852 is an oncogene in BLCA and facilitates the crosstalk between tumour cells and TAMs. The circLOC729852/miR‐769‐5p/IL‐10 axis promotes the recruitment and M2 polarization of TAMs and further facilitates BLCA progression by increasing IL‐10 expression (Figure 7).

## AUTHOR CONTRIBUTIONS

**Changming Dong:** Conceptualization (equal); data curation (equal); formal analysis (equal); methodology (equal); project administration (equal); software (equal); validation (equal); visualization (equal); writing – original draft (equal); writing – review and editing (equal). **Pengyu Hui:** Data curation (equal); formal analysis (equal); funding acquisition (equal); investigation (equal); project administration (equal); validation (equal); writing – review and editing (equal). **Zhengqi Wu:** Data curation (equal); formal analysis (equal); project administration (equal); validation (equal); writing – review and editing (equal). **Jianfeng Li:** Data curation (equal); formal analysis (equal); project administration (equal); validation (equal); writing – review and editing (equal). **Xiaojun Man:** Conceptualization (equal); funding acquisition (equal); investigation (equal); methodology (equal); resources (equal); writing – original draft (equal); writing – review and editing (equal).

## FUNDING INFORMATION

The present study was supported by the China Medical university's 2018 Youth Support Program (natural Science; grant no. QGZ2018041), Scientific Research Project of the Education Department of Liaoning Province (grant no. Qn2019008), the Shenyang Plan Project of Science and Technology (grant no. F19‐112‐4‐098), China Medical University's 2019 discipline Promotion Program, the National Key r&d Plan Key research Projects of Precision Medicine (grant no. 2017YFC0908000) and Xi'an Municipal Bureau of Science and Technology Foundation(22YXYJ0121).

## CONFLICT OF INTEREST STATEMENT

The authors declare that they have no competing interests.

## PATIENT CONSENT FOR PUBLICATION

Not applicable.

## Data Availability

The datasets used during the present study are available from the corresponding author upon reasonable request.
